# A Study on Through-the-Thickness Heating in Continuous Ultrasonic Welding of Thermoplastic Composites

**DOI:** 10.3390/ma14216620

**Published:** 2021-11-03

**Authors:** Bram C. P. Jongbloed, Julie J. E. Teuwen, Rinze Benedictus, Irene Fernandez Villegas

**Affiliations:** Aerospace Structures and Materials Department, Faculty of Aerospace Engineering, Delft University of Technology, Kluyverweg 1, 2629 HS Delft, The Netherlands; J.J.E.Teuwen@tudelft.nl (J.J.E.T.); R.Benedictus@tudelft.nl (R.B.); I.FernandezVillegas@tudelft.nl (I.F.V.)

**Keywords:** fusion bonding, heat transfer, high-frequency welding, joining, CF/PPS, energy director

## Abstract

Continuous ultrasonic welding is a promising technique for joining thermoplastic composites structures together. The aim of this study was to gain further insight into what causes higher through-the-thickness heating in continuous ultrasonic welding of thermoplastic composites as compared to the static process. Thermocouples were used to measure temperature evolutions at the welding interface and within the adherends. To understand the mechanisms causing the observed temperature behaviours, the results were compared to temperature measurements from an equivalent static welding process and to the predictions from a simplified heat transfer model. Despite the significantly higher temperatures measured at the welding interface for the continuous process, viscoelastic bulk heat generation and not thermal conduction from the interface was identified as the main cause of higher through-the-thickness heating in the top adherend. Interestingly the top adherend seemed to absorb most of the vibrational energy in the continuous process as opposed to a more balanced energy share between the top and bottom adherend in the static process. Finally, the higher temperatures at the welding interface in continuous ultrasonic welding were attributed to pre-heating of the energy director due to the vibrations being transmitted downstream of the sonotrode, to reduced squeeze-flow of energy director due to the larger adherend size, and to heat flux originating downstream as the welding process continues.

## 1. Introduction

Because of their high specific mechanical properties, fibre-reinforced polymer composite materials are interesting for industries in which weight is of utmost importance, such as the aerospace industry. The polymers used as matrices in these composite materials are typically thermoset or thermoplastic. Thermoplastics, in contrast to thermosets, do not form a cross-linked network and they rely on the entanglement of linear polymer chains to form a solid structure. These thermoplastic polymer chains become mobile when heated sufficiently above the melting temperature for semi-crystalline polymers or above the glass transition temperature for amorphous polymers. This allows the use of cost-effective manufacturing and joining processes such as press forming and welding for thermoplastic composite parts and structures.

Welding has significant advantages over the more traditional joining methods mechanical fastening and adhesive bonding. For mechanical fastening drilling of holes results in breakage of fibres, and adhesive bonding requires intensive surface treatments and long curing cycles [1]. The most promising welding techniques for thermoplastic composite structures are resistance welding, induction welding, and ultrasonic welding [1,2,3]. The maturity levels of resistance and induction welding are the highest. Consequently, mainly these two technologies are currently being used in the aerospace industry, e.g., the rudder and elevator of the Gulfstream G650 [4] are assembled with induction welding, and the ribs and skin in the fixed leading edge of the A340 and A380 are joined using resistance welding [5]. Many recent studies have been published on process modelling and on experimental investigations to better understand the induction, resistance and ultrasonic welding processes [6,7,8,9,10,11,12,13,14,15], which illustrates the current relevance. Among the three mentioned welding techniques, ultrasonic welding is typically the fastest and lowest energy-consuming joining technique for thermoplastic composites [2]. During the ultrasonic welding process, a static force and high frequency/low amplitude mechanical vibrations are applied to the parts to be welded by means of a sonotrode (vibration phase of the process). Subsequently, the welded joint is allowed to cool down under pressure (consolidation phase). The mechanical vibrations promote the generation of heat through surface and viscoelastic friction [16,17,18]. Heat generation is focused at the weld interface by means of an energy director (ED) placed in between the adherends [17,19]. This ED consists of either one or more resin protrusions moulded on the surface of one of the adherends [20,21,22] or a loose resin-rich layer such as a film (also referred to as flat ED) [19,23] or a woven mesh [14,24] made from the same polymer material as that in the adherends. Due to the lower compressive stiffness of the ED compared to that of the fibre reinforced adherends, the ED undergoes higher cyclic strains and it therefore generates more heat than the adherends [22,25].

**Figure 1:**
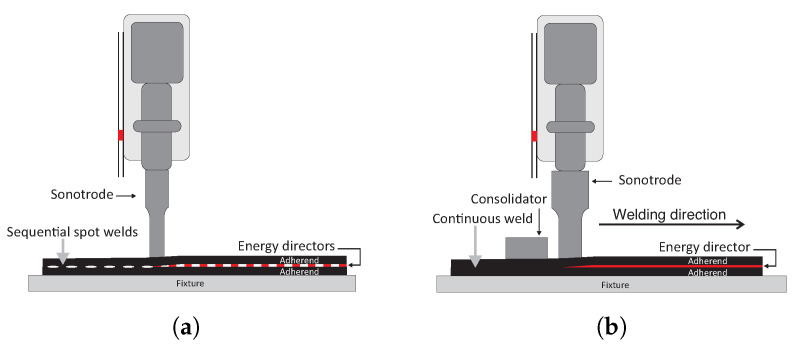
Schematics of static (**a**) and continuous (**b**) ultrasonic welding processes for thermoplastic composites.

**Figure 2:**
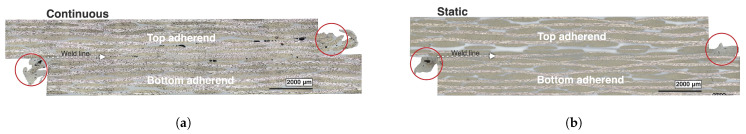
Cross-sectional micrographs of a (**a**) continuous (adapted from [15]) and a (**b**) static ultrasonic weld. Welding force 500 N, vibration amplitude 80 μm, welding speed 35 mm/s (**a**), equivalent vibration time 430 ms (**b**) [14]. The same clamping jig and clamping configuration is used in both cases. The red circles indicate squeeze out of fibres and/or resin.

Ultrasonic welding of thermoplastic composites can be performed in either a static or a continuous fashion. Static ultrasonic welding, e.g., spot welding (Figure 1a) is defined as follows: a relatively small area is welded while both the welder and the adherends remain stationary during the vibration and consolidation phase. During the consolidation phase, the sonotrode itself applies a consolidation pressure. Sequential application of this static process allows obtaining multi-spot welded joints that can match load carrying capabilities of mechanically fastened joints [26,27]. The continuous ultrasonic welding process (Figure 1b) is defined as follows: a relatively large area is welded by continuously translating the welder with respect to the adherends or vice versa while exerting the ultrasonic vibrations and welding pressure [14,24]. Since the sonotrode continuously moves away from the just welded area, an additional consolidation shoe (consolidator) needs to be placed behind the sonotrode to allow the weld to cool down under pressure [15]. The continuous process has some benefits over the static process, i.e., a higher load can be transferred, and the joint is air and liquid tight, its maturity level is however lower. One of the main lessons learned so far in the development of continuous ultrasonic welding of thermoplastic composites is that the compliance of the energy director plays a more prominent role in ensuring weld uniformity than in the static process [24]. Likewise, heating and melting of the energy director extend beyond the footprint of the sonotrode in the continuous welding process and the temperatures at the welding interface are overall higher [14]. Finally, higher through-the-thickness or bulk heating occurs in continuous ultrasonic welding, especially in the adherend in direct contact with the sonotrode. As shown in Figure 2 [15], this leads to significant fibre and resin squeeze out, as well as accompanying porosity, upon application of the consolidation pressure.

Excessive resin and fibre squeeze out are undesirable since they may compromise the structural integrity of the adherends and, hence, of the welded assembly. Consequently, the focus of the present study is gaining a better insight into what causes higher through-the-thickness heating in continuous ultrasonic welding of thermoplastic composites as compared to the static process. In addition, this leads to a better insight into the process and heating mechanisms and thus to high-quality welds. To this end, thermocouples were used to measure the temperature evolution within the adherends and at the weld interface for different welding configurations and combinations of welding parameters. The results were compared to temperature measurements from an equivalent static welding process and to the predictions from a simplified numerical heat transfer model. To further understand the through-the-thickness heating, the total energy input was also reduced by either increasing the welding speed or decreasing the vibration amplitude. Additionally, to understand heating ahead of the sonotrode a damping unit was introduced to dampen vibrations travelling downstream. Finally, the effect of the adherend size, i.e., large adherends for the continuous process versus small coupon sized adherends for the static process, on the temperature evolution was studied by means of static welds on different sized adherends.

## 2. Experimental Procedures

### 2.1. Materials

The thermoplastic composite laminates used for the welding experiments in this study were made out of carbon fibre fabric (five harness satin weave) impregnated with polyphenylene sulphide powder, CF/PPS semipreg (CF 0286 127 Tef4 43% from Toray Advanced Composites, Nijverdal, The Netherlands). The laminates were stacked according to a ${\left[0/90\right]}_{3s}$ sequence and subjected to a consolidation process in a hot platen press for 20 min at 320 ${}^{\circ }$C and 1 MPa pressure, which, based on previous experience, results in void-free laminates. The consolidated laminates had a size of 580 mm by 580 mm and a thickness of approximately 1.85 mm. Different size adherends measuring 220 mm × 101.6 mm and 15 mm × 101.6 mm were cut from the consolidated laminates using a water jet cutter. For both adherend sizes, the main apparent fibre orientation was in the 101.6 mm direction. A 0.20 mm thick woven PPS mesh energy director with 37% open area (PPS100, supplied by PVF GmbH, Markt Schwaben, Germany) was used in all experiments to focus heat generation at the welding interface [14,24].

### 2.2. Continuous Ultrasonic Welding

**Figure 3:**
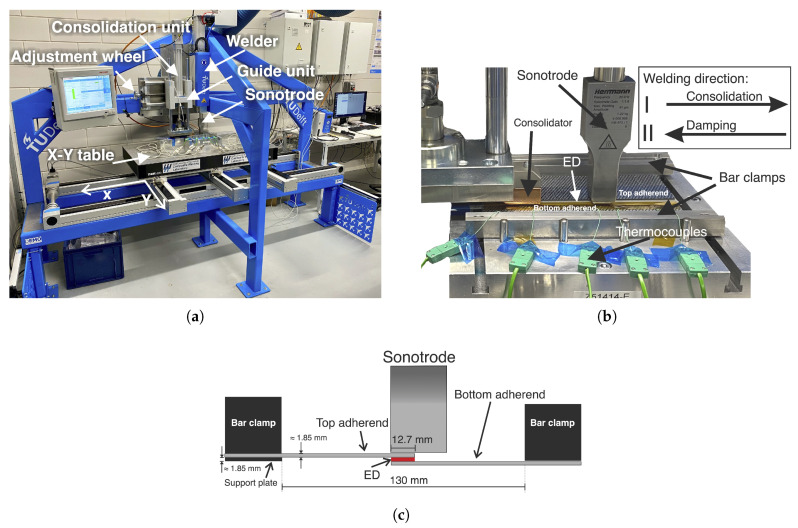
(**a**) Custom-built welding machine for static and continuous ultrasonic welding, (**b**) close-up of the continuous welding set-up visualising relative sonotrode and consolidator placement, and (**c**) schematic side-view of clamping distance and sonotrode placement.

The custom-built ultrasonic welding machine shown in Figure 3a was used for the continuous ultrasonic welding experiments. It consists of a stiff frame with an X-Y table on a guiding system, an off-the-shelf ultrasonic welder (VE20 SLIMLINE DIALOG 6200, Herrmann Ultrasonics, Karlsbad, Germany), and a custom-built consolidation unit. The welder vibrates at a fixed frequency of 20 kHz. Both the welder and consolidation unit are connected to the stiff frame. A rectangular sonotrode with a 15 mm × 27 mm contact area and a maximum peak-to-peak operational amplitude of 80 μm was used in the welding setup. The consolidation unit consisted of a 1.5 kN servo press kit (YJKP, Festo, Delft, The Netherlands), a stabilization guide unit to avoid sideways deflections, and a 40 mm × 30 mm copper block (Figure 3b). The consolidation force could be adjusted to a maximum of 1500 N. The distance between the consolidator and the sonotrode was set to 86.4 mm, based on the results of our previous study [15]. During the welding process, the adherends were translated with respect to the sonotrode generally following the welding direction I (Figure 3b), while the sonotrode continuously exerted the static welding force and vibrations, and the consolidator applied pressure. For some experiments, the consolidation unit was used as a damping unit for which it was placed 18.4 mm ahead of the sonotrode (Figure 3b) and welding took place in the opposite direction (II). An aluminium base with bar clamps was used to clamp the adherends during the welding process (Figure 3b). Figure 3c shows the positioning of the bar clamps and of the sonotrode relative to the overlap. Note that this configuration, which we found to provide a more uniform temperature distribution across the overlap [15], differs slightly from the one used in [14]. The different sets of welding parameters used in this study are shown in Table 1.

### 2.3. Static Ultrasonic Welding

The same experimental setup as described in the previous subsection (Figure 3) was used for the static welding process. In this case, however, the adherends remained stationary and the consolidation unit was not used. The welding parameters used in the static process are shown in Table 1. The welder was set to vibrate for 430 ms, after which the vibrations stopped (end of vibrations), and the consolidation phase was initiated. As mentioned in the introduction, in the static process the sonotrode also provides the consolidation pressure during the consolidation phase. Note that the vibration time used for the static process, 430 ms, is equivalent to 35 mm/s welding speed in a continuous welding process with a 15 mm wide sonotrode. Static welds on the 15 mm wide adherends covered the entire overlap, whereas, in the case of 220 mm wide adherends, a single 15 mm wide static weld was created in the middle of the 220 mm wide overlap. During the static welding process, the vertical position of the sonotrode was monitored at a 1 kHz sampling frequency.

### 2.4. Temperature Measurements

**Figure 4:**
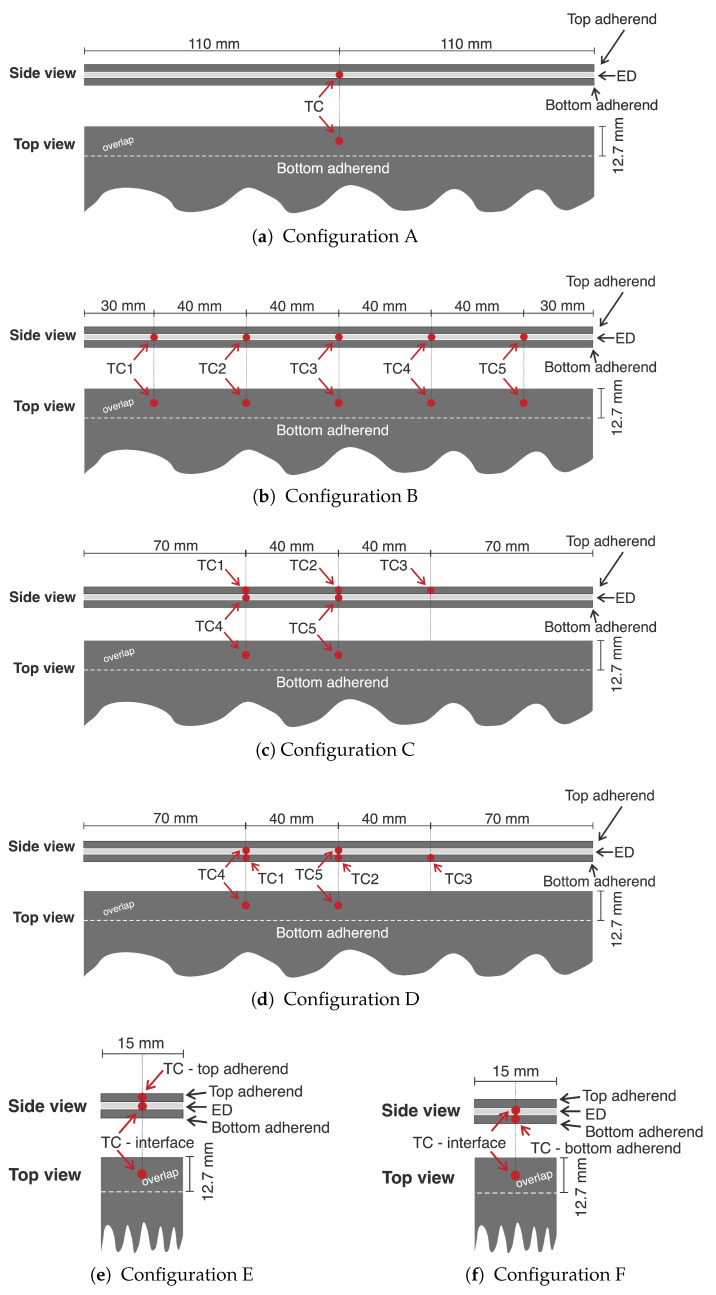
Schematic side and top view of the temperature measurement configurations used in this study on 220 mm wide and 15 mm wide adherends.

Temperatures were measured at the welding overlap and within the adherends using K-type thermocouples (GG220-2K-0, product number 2-2200-0004, Tempco B.V., Bodegraven, the Netherlands). The sleeved thermocouples had a total diameter of 0.70 mm, while the diameter of the thermocouple wires was 0.10 mm. An analogue thermocouple output amplifier (Adafruit AD8495) was used to simultaneously sample temperature readings at 1 kHz from a maximum of five thermocouples. A moving average filter (10 points for the static process and 25 or 60 points for the continuous process) was applied in MATLAB to filter out high-frequency fluctuations from the temperature data. For the temperature measurements at the weld interface, the thermocouples were placed in the middle of the overlap, sandwiched between the bottom adherend and the energy director. For the measurements within the adherends, the thermocouples were inserted into 0.7 mm diameter holes drilled up to a depth of approximately 7 mm. The placement and depth of each hole ensured that the tip of the thermocouple was located approximately in the middle of the overlap width (directly above or below the thermocouple at the weld interface) and approximately midway through the thickness of the adherend. Note that the diameter of the hole and of the sleeved thermocouple were the same to ensure a press fit. To measure the temperature at the weld interface and through the thickness in the top or bottom adherends, different thermocouple configurations shown in Figure 4 were used for the static (configurations A, E, and F) and continuous welding process (configurations B, C and D). Table 1 shows which of these thermocouple configurations were used in the different experiments.

### 2.5. Testing and Analysis Techniques

Whenever necessary, the continuously welded adherends were cut into six 25.4 mm wide single lap shear samples with a diamond saw of which five were used for mechanical testing. The remaining sample was used for cross-sectional microscopy. The 28.8 mm wide edges at the start and at the end of the continuous welds were discarded. The single-lap shear samples were mechanically tested with a Zwick/Roell 250 kN (Kennesaw, GA, USA) universal testing machine with a cross-head speed of 1.3 mm/min. The grips were given the necessary offset to minimize secondary bending. The apparent lap shear strength (LSS) was calculated by dividing the maximum load by the overlap area. After mechanical testing, a non-contact roughness measurement/profiler system (Keyence VR-5000, Mechelen, Belgium) was used for the analysis of fracture surfaces. To obtain cross-sectional views from the welded adherends, specimens were cut and embedded in epoxy resin. They were ground and polished with a Struers Tegramin-20 polisher (Ballerup, Denmark). A 3D laser scanning confocal microscope (Keyence VK-X1000, Mechelen, Belgium) was used for inspecting the cross-sections.

### 2.6. Heat Transfer Model

A 2D transient heat transfer model representing the static welding process was created in COMSOL Multiphysics 5.5 (Burlington, MA, USA) to estimate the temperature increase in the adherends resulting from only heat conduction from the weld interface in both the static and the continuous process. Since the energy director is very thin compared to the adherends, it was not included in the model. The heat transfer model is based on the following heat transfer equation:

(1) $$\rho C_{p}\frac{\partial T}{\partial t}=k\nabla^{2}T$$

in which *T* is temperature, *t* is time, and ρ, $C_{p}$, and *k* are the density, thermal capacity, and thermal conductivity of CF/PPS, respectively.

**Figure 5:**
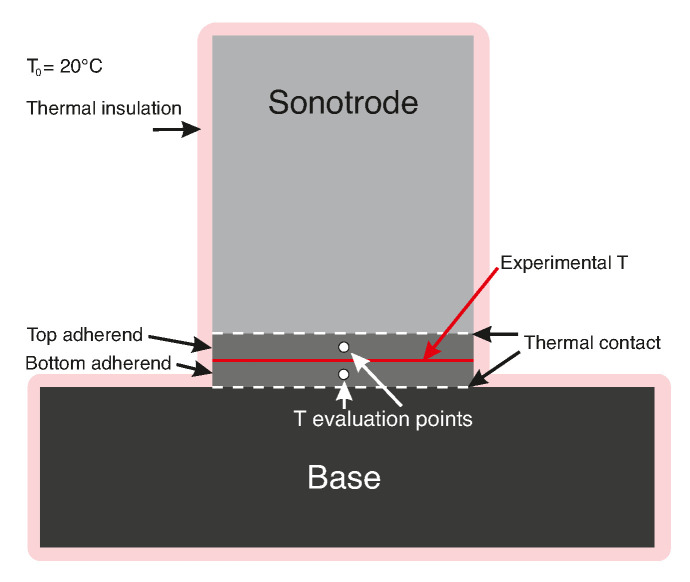
Schematic model with boundary conditions for heat transfer model of static welding setup.

Figure 5 shows the geometry and boundary conditions and Table 2 lists the material properties used in the model as per Equation (1). The outer boundaries were thermally insulated and the initial temperature of the sonotrode and base was 20 ${}^{\circ }$C. The experimental temperature data obtained from measurements at the welding interface was applied as an input to the entire welding interface in the model. The resulting temperature evolution predicted by the model was evaluated midway through the thickness of the top and of the bottom adherends. It should be noted that due to the conservative nature of the boundary conditions (i.e., no heat dissipation to the environment) as well as the room-temperature material properties used for the CF/PPS material, the model most likely overestimates the heat transferred from the weld interface to the adherends and hence higher overall temperatures when used for the static process. Nevertheless, by comparing the temperature evolution provided by the model and the experimental value, the simplified model is still a valuable tool to discern whether the cause of through-the-thickness heating in the adherends is heat transfer or there are other sources. When used for the continuous process, however, the following simplifications in the model: (i) the sonotrode is in contact with the top adherend during the entire cooling phase, and (ii) there is no heat flux from downstream and upstream of the sonotrode into the studied area, can be expected to somewhat offset this overestimation. It should be noted that the model is not suitable for accurately predicting the actual temperature values, since that would require a 3D model with temperature-dependent material properties and in case of the continuous process a more complicated dynamic model with a moving welder head.

## 3. Results

**Figure 6:**
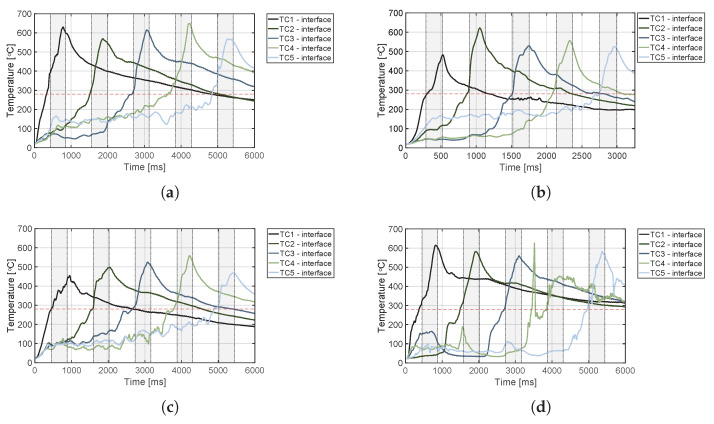
Temperature evolution for the continuous welding process at the weld interface for: (**a**) reference case, (**b**) higher welding speed case (65 mm/s), (**c**) lower amplitude case (70 μm), and (**d**) damping case. TC1 to TC5 were respectively located under the sonotrode during the five grey areas. The red dashed line indicates the melting temperature of PPS ($T_{m}$, 280 ${}^{\circ }$C) as experimentally determined by DSC analysis.

**Figure 7:**
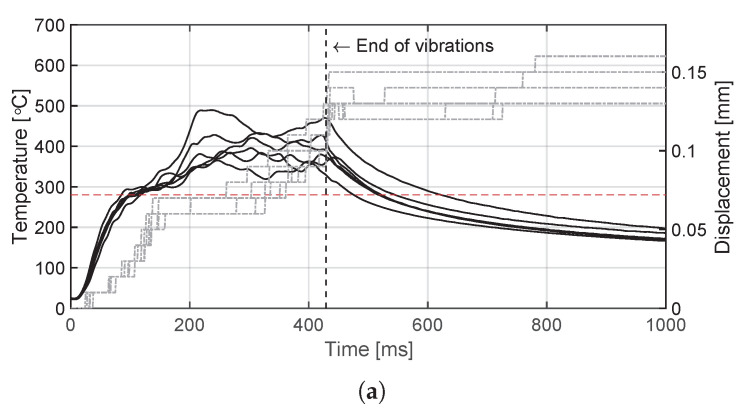

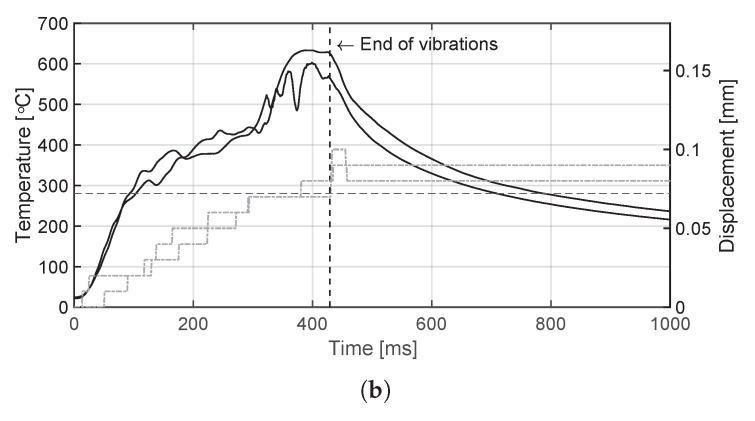
Interface temperature (black curves) and measured downward vertical displacement (grey curves) of the sonotrode for static welds on (**a**) 15 mm wide and (**b**) 220 mm wide adherends. It should be noted that one of the two welds in (**b**) was made 40 mm to the left from the intended location in Figure 4a. The red dashed line indicates the melting temperature of PPS ($T_{m}$, 280 ${}^{\circ }$C) as experimentally determined by DSC analysis.

**Figure 8:**
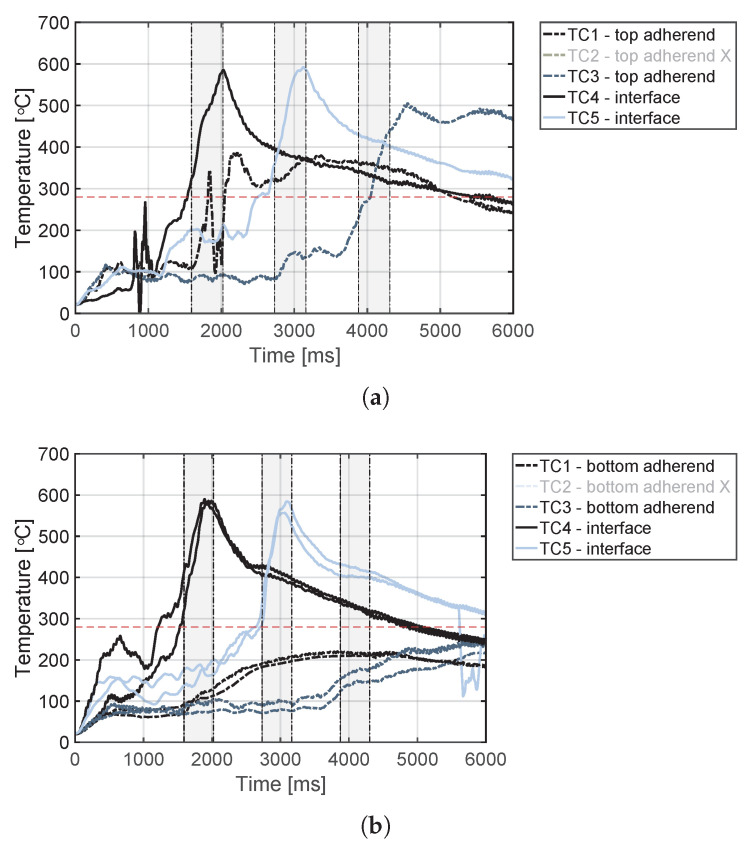
Temperature evolution for the continuous welding process at the weld interface and through the thickness for (**a**) the top adherend and (**b**) the bottom adherend. The grey areas indicate the time span during which a specific thermocouple was located under the sonotrode. The red dashed line indicates the melting temperature of PPS ($T_{m}$, 280 ${}^{\circ }$C) as experimentally determined by DSC analysis.

**Figure 9:**
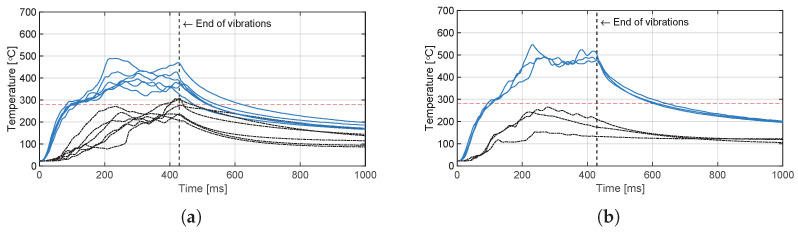
Temperature evolution for the static welding process at the weld interface (blue curves) and through the thickness (black curves) for (**a**) the top adherend and (**b**) the bottom adherend. The temperature evolution at the weld interface in (**a**) is the same as Figure 7a. The red dashed line indicates the melting temperature of PPS ($T_{m}$, 280 ${}^{\circ }$C) as experimentally determined by DSC analysis.

**Figure 10:**
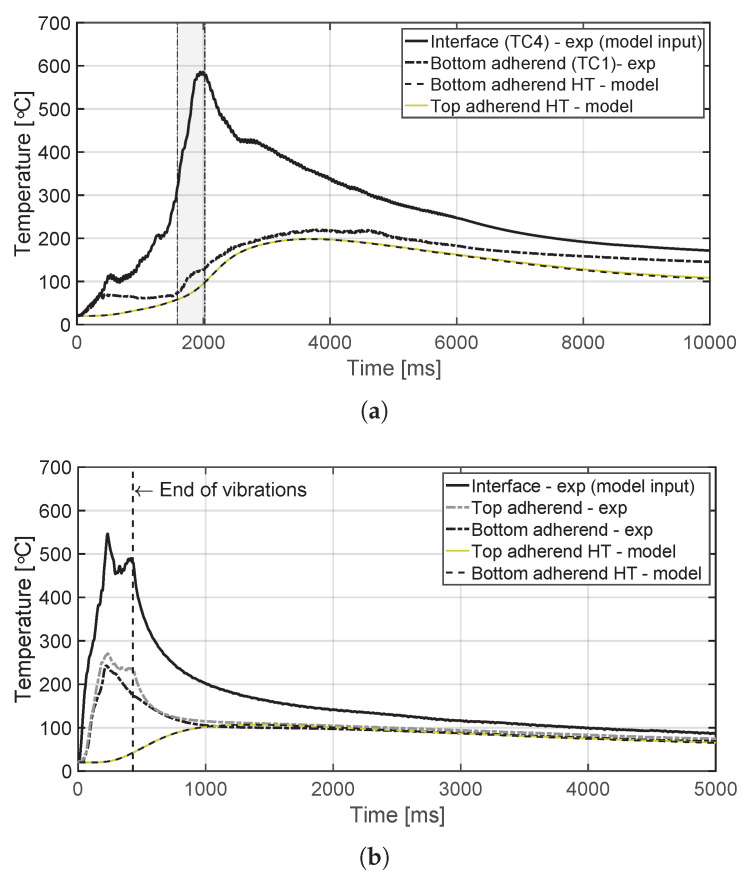
Modelled through-the-thickness temperature evolution due to heat transfer (HT) as a result from experimental (exp) temperature input at the weld interface for (**a**) the continuous and (**b**) static temperature evolution together with a representative experimental temperature evolution as a reference. The grey area (**a**) and the vertical line (**b**) indicate the time span during which the thermocouples were located under the sonotrode. Note that in (**a**) no experimental temperature evolution for the top adherend is shown as it was deemed less trustworthy due to severe overheating.

**Figure 11:**
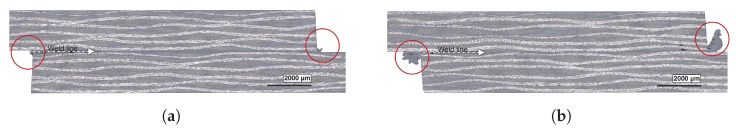
Representative cross-sectional micrographs of continuous ultrasonic welds for (**a**) higher speed case (65 mm/s), and (**b**) lower amplitude case (70 μm). Red circles indicate squeeze-out location.

**Figure 12:**
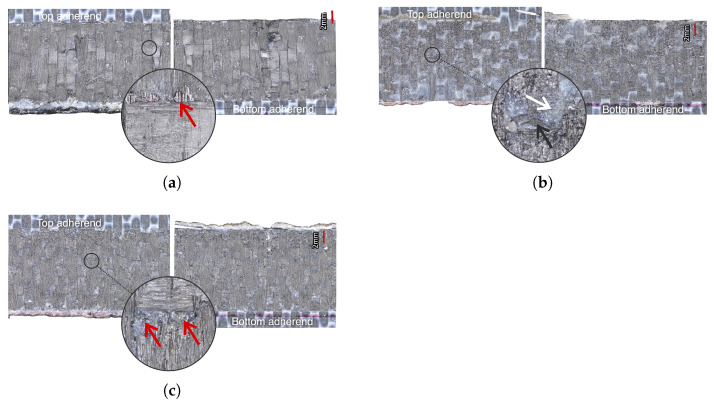
Representative fracture surfaces from continuous welds for (**a**) the reference case, (**b**) higher welding speed case (65 mm/s), and (**c**) the lower amplitude case. Red arrows indicate voids, white arrow indicates unwelded area together with voids, and black arrow indicates area without connection between top and bottom adherends.

The temperature evolution at the weld interface for the continuous process is shown in Figure 6 for the reference case, higher welding speed case, lower amplitude case, and consolidator as damping case (Figure 6a, Figure 6b, Figure 6c, and Figure 6d, respectively). Figure 7 shows temperature and vertical sonotrode displacement curves for static welds on (a) 15 mm wide and (b) 220 mm wide adherends. Figure 8 and Figure 9 show the temperature evolution at the weld interface and through the thickness for the (a) top and (b) bottom adherends in the continuous and static process, respectively. It should be noted that some thermocouples malfunctioned during the continuous process (Figure 4c,d): TC2 for all welds and multiple thermocouples in one weld for configuration D (Table 1). Therefore, these results have been omitted. The measurements within the top adherend (Figure 8a) might be less trustworthy as severe overheating was observed of this adherend at the thermocouple locations and thermocouples TC3 and TC4 partially malfunctioned (as seen by the temperature spikes in Figure 8a). Figure 10 shows the modelled temperature prediction due to heat transfer to the middle of the adherends for the static welding setup (indicated in Figure 5) based on the experimental temperature input at the weld interface from (a) the continuous and (b) the static welding process. It should be noted that for the top adherend in the continuous process no experimental value is shown because, as mentioned before, the top adherend was severely overheated at the thermocouple locations making the results less trustworthy. Figure 11 shows cross-sectional micrographs from continuous ultrasonic welds for (a) the higher speed case and (b) the lower amplitude case. Note that for the reference case the cross-section is shown in Figure 2a. Representative fracture surfaces from the reference, higher speed, and lower amplitude cases are shown in Figure 12a, Figure 12b, and Figure 12c, respectively. The single-lap shear strength values for the continuous and static welds are shown in Table 3.

## 4. Discussion

The aim of this study was gaining further insight into what causes higher through-the-thickness heating in continuous than in static ultrasonic welding. Through-the-thickness heating combined with the application of the welding/consolidation pressure may cause matrix and fibre squeeze flow as well as porosity in the adherends [15] and hence may affect their structural integrity. Firstly, the heating mechanisms responsible for through-the-thickness heating were investigated by comparing temperature measurements from the static and continuous process to heat transfer model predictions. Secondly, it was studied what causes the temperature differences between the static and continuous process. Finally, the quality of the welds was discussed in view of the temperature evolution.

**Figure 13:**
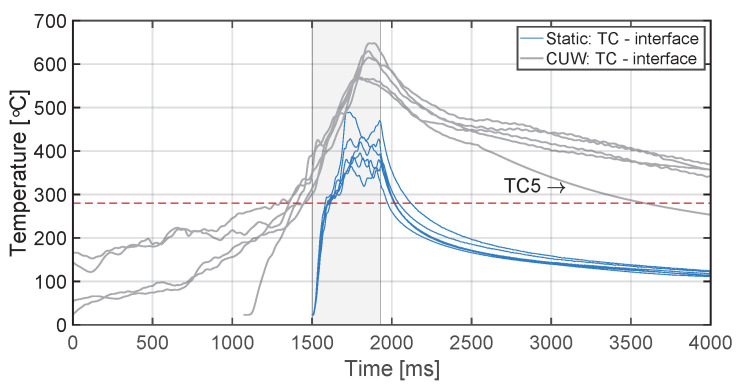
Combined interface temperature evolutions of continuous (reference case) (Figure 6a) and static (Figure 9a) processes superimposed for time that thermocouple experiences vibrations directly under sonotrode. The red dashed line indicates the melting temperature of PPS as experimentally determined by DSC analysis.

A plausible cause of higher through-the-thickness heating is the higher overall temperatures at the welding interface (Figure 13) resulting in increased thermal conduction to the adherends. The predictions of the heat transfer model (Figure 10) show indeed that the higher interface temperatures in the continuous process do result in higher temperature increase caused by conduction in the middle of the adherends. However, there are notorious differences between measured and predicted temperature evolutions for the adherends in the static process (Figure 10b) and presumably for the top adherend in the continuous process (Figure 8a and Figure 10a). These differences indicate the existence of an extra heating mechanism responsible for the steep temperature increase of the adherends during the heating phase of the welding process (Figure 8a and Figure 9) in addition to the relatively gentle temperature increase caused by thermal conduction observed in the model predictions (Figure 10), which peaks during the cooling phase. This extra mechanism is bulk viscoelastic heating and its prevalent role in the temperature evolution in the adherends is not surprising since, once compressed, the energy director is very thin (≈0.10 mm) and hence it has a low compliance [24] and low ability to concentrate viscoelastic heat generation at the interface only [23].

Contrarily, temperature measurements in the bottom adherend during the continuous process (Figure 8b) show trends that are closer to what one could expect from thermal conduction from the interface (Figure 10a). This observation, together with the overheating observed at the locations of the thermocouples within the top adherend (Figure 8a), prompts us to think that in the continuous process the top adherend absorbs significantly more vibration energy than the bottom adherend. This is contrary to a more balanced energy distribution in the static process (Figure 9). This is consistent with the top adherend experiencing more severe heating than the bottom adherend and than any of the adherends in the static process under equivalent process parameters (Figure 2). Reducing the total vibration energy associated with the process, by either increasing the welding speed or decreasing the vibration amplitude, has indeed a substantial effect on reducing through-the-thickness heating and the corresponding matrix and fibre squeeze flow in the top adherend (Figure 11) supporting our hypothesis. The cause of the uneven distribution of vibration energy between the top and bottom adherend in the continuous process is yet unknown. We believe that the translation of the vibrating sonotrode on the surface of the top adherend might cause an extra component of cyclic strains parallel to the welding interface resulting in a superposition of viscoelastic heating sources in that adherend. Further research should however be performed to test this hypothesis.

Additionally, given the importance of the temperature evolution at the welding interface in the quality of the welded joints, it is important to understand what causes the temperature differences between the continuous and the static process (Figure 13) and their relevance in the outcome of the welding process. It should be noted that the temperature differences between the two processes shown in Figure 13 are much higher than those reported in our previous work [14]. We believe such seeming inconsistency stems from the different clamping schemes in both studies; in particular differences in the clamping distance, which is known to affect the cycling strains and, hence, heat generation in the energy director [29].

**Figure 14:**
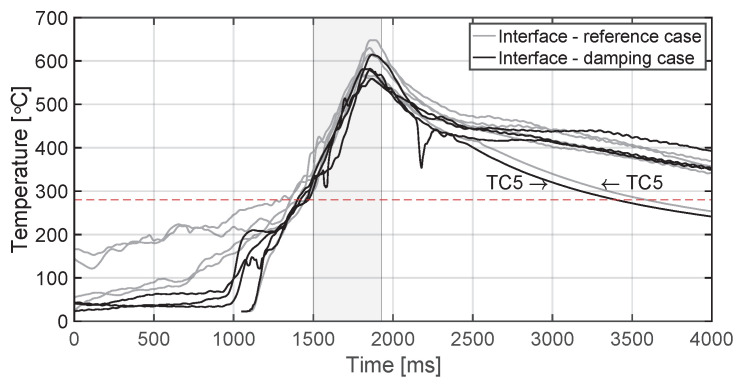
Combined interface temperature evolutions of the continuous ultrasonic welding process for reference case (Figure 6a) and damping case with the consolidator as damping unit (Figure 6d without TC4) superimposed for time that thermocouple experiences vibrations directly under sonotrode (grey area). The red dashed line indicates the melting temperature of PPS as experimentally determined by DSC analysis.

Firstly, the pre-heating experienced by the energy director, which is consistent with previous observation of thermal effects in the energy director downstream of the sonotrode [14], is believed to result from the ultrasonic vibration being transmitted downstream of the sonotrode. Far from the sonotrode, surface friction between thermocouples, energy director and adherends most likely causes the temperature to almost instantaneously increase to around 100 ${}^{\circ }$C at the onset of the welding process. Once the sonotrode is close enough for the stiff adherends to exert sufficient pressure and, hence, sufficient cyclic strain in the energy director, viscoelastic heating accounts for a faster temperature increase. Indeed, damping of the vibrations beyond the sonotrode effectively minimizes pre-heating far from the sonotrode (Figure 14). It does not, however, remove pre-heating as the sonotrode gets closer to the measuring location owing to limitations in the experimental setup (i.e., the minimum practical distance between the damping unit and the sonotrode).

Secondly, the temperature at the interface experiences a continuous increase during the time the sonotrode is moving above a certain location which results in higher maximum temperatures as compared to the static process (Figure 13). Based on the results of the static welds on the same adherend size used for the continuous process (Figure 7), this continuous temperature increase can be attributed to the limitations imposed by the adherends to the squeeze flow of the energy director (as seen in a lower total displacement) and, consequently, limitations to the associated cooling effect (as seen in the continuous temperature increase) [23].

**Figure 15:**
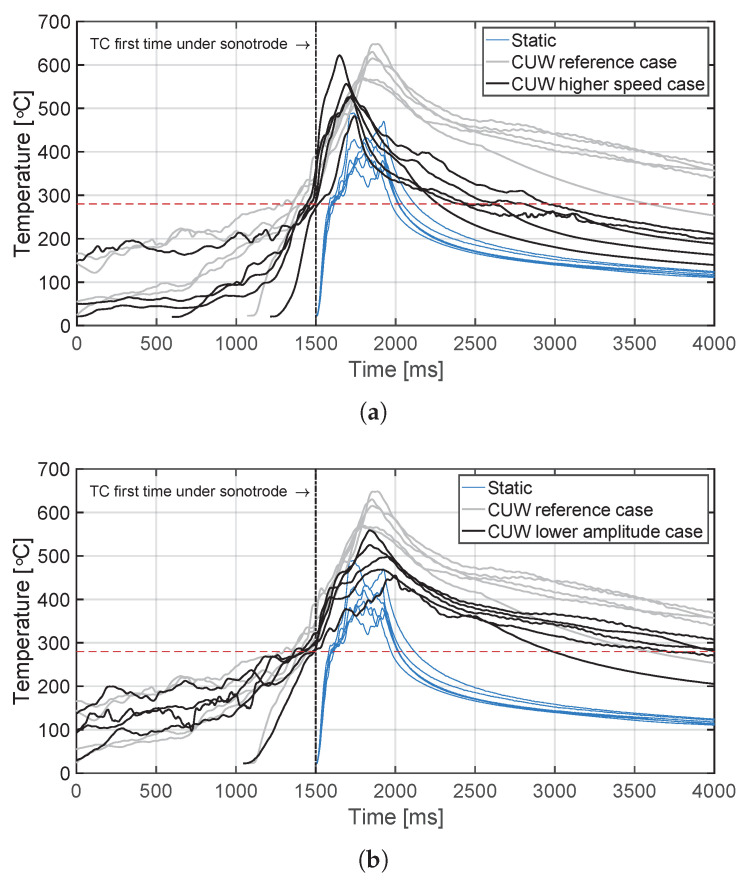
Combined interface temperature evolutions of the reference case (Figure 6a) of the continuous process, the static process (Figure 9a), and the temperature evolutions for (**a**) the higher welding speed case (65 mm/s) (Figure 6b), and (**b**) the lower amplitude case (70 μm) (Figure 6c). The temperature evolutions were superimposed at the moment they were under the sonotrode for the first time. The red dashed line indicates the melting temperature of PPS as experimentally determined by DSC analysis.

Thirdly, longer times needed to cool down the interface below T${}_{m}$ are associated with the heat flux originated downstream as the welding process progresses, as indicated by the consistently faster cooling measured by the last thermocouple (TC5) in the weld line (i.e., the one with the lowest influence by downstream heating, Figure 13 and Figure 14). It is worth noticing that before this effect becomes relevant (i.e., beyond divergence point between thermocouple measurements in Figure 13 and Figure 14), there is an initial faster cooling stage most likely influenced by the temperature in the composite layers in close proximity to the interface. In fact, the process that results in the lowest matrix and fibre squeeze out from the top adherend, i.e., higher speed case, is the one showing a significantly higher cooling rate in that initial stage (Figure 15a).

Finally, regarding the quality of the welds, it is interesting to note that in those cases in which the strength of continuously welded joints is comparable (or higher) to that of the static welds (i.e., reference and lower amplitude case, see Table 3), the average area delimited by the temperature curves and the melting temperature of PPS (presumably related to the actual thermal energy invested in the creation of the welded joints) is much higher than that in the static process (Figure 13 and Figure 15b). Interestingly, in the case in which both areas are closer to each other (i.e., higher speed case, Figure 15a) the strength of continuous joints is significantly lower in accordance with the presence of unwelded areas on the fracture surfaces (Figure 12b). These observations, which indicate that in the continuous process the thermal energy input required to create a weld is higher than in the static process, relate to the lower temperature in the bottom adherend (as discussed previously) hindering the creation of the welded joint.

## 5. Conclusions

The aim of this study was to gain further insight into what causes higher through-the-thickness heating in continuous ultrasonic welding of thermoplastic composites as compared to the static process. In the continuous process, temperatures at the welding interface were indeed found to be significantly higher than in the static process. This was attributed to a combination of pre-heating of the energy director due to the vibrations being transmitted downstream of the sonotrode, reduced squeeze-flow of the energy director due to the larger adherend size, and heat flux originating downstream as the welding process continues. Thermal conduction from the hotter interface was however found to not be the main cause of higher through-the-thickness heating in the top adherend, which was in turn attributed to viscoelastic bulk heat generation. The top adherend seemed to indeed absorb most of the vibrational energy in the continuous process as opposed to a more balanced energy share between the top and bottom adherend in the static process. On the contrary, the bottom adherend showed a temperature evolution similar to what could be expected from predominant thermal conduction from the welding interface. Consequently, reducing the total vibration energy introduced in the material in the continuous welding process proved to have a substantial effect on reducing through-the-thickness heating.

## Figures and Tables

**Table 1:** Overview of Continuous (CUW) and Static (SUW) Ultrasonic Welding Experiments (Note: the amplitude (amp) values are peak-to-peak).

Process	Welding Parameters (Speed/Time, Force, Amp)	Consolidation Parameters	Number of Welds	Adherend Size (mm)	Config (Figure 4)	Remarks
**CUW**	35 mm/s, 500 N, 80 μm	800 N (1.6 MPa)	1	220 × 101.6	B	Setup with consolidation unit (Figure 3b). Reference case.
	35 mm/s, 500 N, 80 μm	800 N (1.6 MPa)	1	220 × 101.6	C	Setup with consolidation unit (Figure 3b).
	35 mm/s, 500 N, 80 μm	800 N (1.6 MPa)	3	220 × 101.6	D	Setup with consolidation unit (Figure 3b).
	35 mm/s, 500 N, 80 μm	800 N (1.6 MPa)	1	220 × 101.6	B	Setup with consolidation unit as damping unit (Figure 3b).
	65 mm/s, 500 N, 80 μm	800 N (1.6 MPa)	1	220 × 101.6	B	Setup with consolidation unit (Figure 3b). Higher speed case.
	35 mm/s, 500 N, 70 μm	800 N (1.6 MPa)	1	220 × 101.6	B	Setup with consolidation unit (Figure 3b). Lower amp case.
**SUW**	430 ms, 500 N, 80 μm	300 N (1.6 MPa) for 4 s	6	15 × 101.6	E	-
	430 ms, 500 N, 80 μm	300 N (1.6 MPa) for 4 s	3	15 × 101.6	F	-
	430 ms, 500 N, 80 μm	300 N (1.6 MPa) for 4 s	2	220 × 101.6	A	Static weld at TC location

**Table 2:** Material Properties used for Model.

CF/PPS Adherends		
**Property**	**Value**	**Remarks**
Density (ρ)	1540 kg/m${}^{3}$	Calculated based on weight measurements
Heat capacity $\left(C_{p}\right)$	681 J/(kg$\cdot^{\circ }$C)	Value taken at 20 ${}^{\circ }$C [28]
Thermal conductivity (k)	0.34 W/(m$\cdot^{\circ }$C)	Value taken at 20 ${}^{\circ }$C [28]
**Aluminum Base**		
**Property**	**Value**	**Remarks**
Density (ρ)	2665 kg/m${}^{3}$	From COMSOL material library of aluminum 5083
Heat capacity $\left(C_{p}\right)$	955 J/(kg$\cdot^{\circ }$C)	From COMSOL material library of aluminum 5083 Value shown at 20 ${}^{\circ }$C
Thermal conductivity (k)	120 W/(m$\cdot^{\circ }$C)	From COMSOL material library of aluminum 5083 Value shown at 20 ${}^{\circ }$C
**Steel Sonotrode**		
**Property**	**Value**	**Remarks**
Density (ρ)	7860 kg/m${}^{3}$	From COMSOL material library of steel 1040
Heat capacity $\left(C_{p}\right)$	480 J/(kg$\cdot^{\circ }$C)	From COMSOL material library of steel 1040 Value shown at 20 ${}^{\circ }$C
Thermal conductivity (k)	52 W/(m$\cdot^{\circ }$C)	From COMSOL material library of steel 1040 Value shown at 20 ${}^{\circ }$C

**Table 3:** Average Lap Shear Strength (LSS) Values with Standard Deviation for Different Continuous and Static Welds.

Process	Parameters (Speed/Time, Force, Amplitude)	LSS (MPa)	Remarks
CUW	35 mm/s, 500 N, 80 μm	39.6 ± 2.3 (*n* = 5)	Reference case. Samples tested from configuration B with consolidation unit (Figure 3b).
CUW	35 mm/s, 500 N, 70 μm	37.2 ± 2.5 (*n* = 5)	Lower amplitude case. Samples tested from configuration B with consolidation unit (Figure 3b).
CUW	65 mm/s, 500 N, 80 μm	25.0 ± 3.9 (*n* = 5)	Higher speed case. Samples tested from configuration B with consolidation unit (Figure 3b).
SUW	440 ms, 500 N, 80 μm	34.3 ± 1.2 (*n* = 4)	Value taken from [14].

## Data Availability

The data presented in this study are available on request from the corresponding author.
